# Splenic-preserving total duodenopancreatectomy and a conservative postoperative management: a case report

**DOI:** 10.1093/jscr/rjag858

**Published:** 2026-09-28

**Authors:** Khadija El Aalaoui, Sarah Benammi, Nsiri Zakaria, Achour Youssef, Ezzaki Houda, Benkabbou Amine

**Affiliations:** Faculty of Medicine of Rabat, Mohammed VI University of Sciences and Health, Morocco; Faculty of Medicine of Rabat, Mohammed VI University of Sciences and Health, Morocco; International University Hospital Mohammed VI, Rabat, Morocco; Faculty of Medicine of Rabat, Mohammed VI University of Sciences and Health, Morocco; International University Hospital Mohammed VI, Rabat, Morocco; Faculty of Medicine of Rabat, Mohammed VI University of Sciences and Health, Morocco; International University Hospital Mohammed VI, Rabat, Morocco; Faculty of Medicine of Rabat, Mohammed VI University of Sciences and Health, Morocco; International University Hospital Mohammed VI, Rabat, Morocco; Faculty of Medicine of Rabat, Mohammed VI University of Sciences and Health, Morocco; International University Hospital Mohammed VI, Rabat, Morocco

**Keywords:** pancreatic ductal adenocarcinoma, spleen-preserving total duodenopancreatectomy, total duodenopancreatectomy (TDP)

## Abstract

Pancreatic ductal adenocarcinoma (PDAC) is aggressive. Tumors in the isthmus or body may require extended resection. Splenectomy is common, but spleen preservation can reduce hematologic and infectious morbidity while risking splenic ischemia. A 65-year-old man with insulin-treated type 2 diabetes and mid-body PDAC underwent neoadjuvant chemotherapy and laparoscopic spleen-preserving total duodenopancreatectomy. The patient was admitted in the intensive care unit on postoperative day 1 and 2. On postoperative day 9 patient reported left upper-quadrant pain; computed tomography scan showed a small splenic infarct managed nonoperatively with analgesia and a short antibiotic course. Discharged day was on postoperative day 16. Final pathology reported moderately differentiated PDAC with high-grade PanIN, lymphovascular, and perineural invasion, peripancreatic fat infiltration; 6/26 nodes were positive (ypT3 N2) and 6 nodes with non-necrotizing granulomas. Margins were negative. Spleen-preserving total duodenopancreatectomy can achieve oncologic clearance but poses technical challenges and splenic ischemia risk that can be managed with non-invasive approach. Feasible for select centrally located PDACs with meticulous vascular technique and vigilant follow-up.

## Introduction

Pancreatic ductal adenocarcinoma (PDAC) is the most common and aggressive exocrine pancreatic malignancy, arising from the pancreatic ductal epithelium and characterized by early local invasion, nodal spread, and resistance to therapy [1]. Tumors centered in the isthmus/body that extend to adjacent organs often require extended resection to achieve negative margins [2]. A total duodenopancreatectomy, even when performed laparoscopically, is a major procedure with high morbidity (hemorrhage, intra-abdominal infection, sepsis, prolonged hospitalization, reoperation, pulmonary, and thrombo-embolic complications). Concomitant splenectomy increases infectious and thrombotic risks (including overwhelming post-splenectomy infection and reactive thrombocytosis) and requires specific preventive measures (vaccination, patient education, tailored surveillance). Spleen preservation may reduce these risks by maintaining immune function but adds technical difficulty and carries a non-negligible risk of splenic ischemia, infarction, abscess or secondary splenectomy. The choice must be individualized, balancing oncologic safety, vascular involvement, comorbidity, and perioperative resources in multidisciplinary discussion. We present a case of an isthmo-corporeal PDAC treated with a spleen-preserving total duodenopancreatectomy.

## Case presentation

A 65-year-old man with type 2 diabetes presented with progressive epigastric pain, weight loss, and new-onset steatorrhea. Imaging and endoscopic workup confirmed an isthmo-corporeal pancreatic mass consistent with PDAC: multiphase pancreatic-protocol computed tomgoraphy (CT) scan including arterial and portal venous phase, magnetic resonance imaging, endoscopic ultrasound with biopsy, and staging positron emission tomography/CT. Tumor makers CA 19–9 and angiotensin-converting enzyme were not significant. He received neoadjuvant therapy and underwent a total duodenopancreatectomy with spleen preservation. Perioperative findings included total pancreatectomy and ligation section of the splenic artery due to the close contact with the tumor to complete resection and negative margins. He spent two nights in the ICU and developed a postoperative splenic infarct presenting as left upper-quadrant pain radiating to the left scapula, managed conservatively. Abdominal CT-scan with contrast on postoperative day (POD) 4 showed splenic infarct associated with closed collection (Fig. 1). Upon concertation with interventional team (IR) decision was made for conservative management considering clinical tolerance. Analgesia and oral antibiotics were introduced for management of non-complicated sepsis associating pain and resolving fever. He was discharged on postoperative day 16 after clinical and biological recovery. Histopathology showed residual moderately differentiated pancreatic ductal (canalicular) adenocarcinoma with associated high-grade PanIN, lymphovascular emboli, perineural invasion, and peri-pancreatic fat infiltration; all margins were negative. Six of 26 lymph nodes were positive (largest metastasis 7 mm) with one capsular breach (ypT3 N2 Mx) and College of American Pathologists Tumor Regression Grade 2 (CAP TRG 2). Six additional nodes demonstrated non-necrotizing epithelioid and giant-cell granulomatous inflammation; an etiologic workup was recommended. The gallbladder (8 × 3 cm) showed chronic cholecystitis without malignancy. Adjuvant chemotherapy was started at 6 weeks postoperatively, after clinical recovery and radiologic evidence of resolving splenic ischemia. Follow-up at 6 month was uneventful with unremarkable clinical status and stationary tumor markers.

**Figure 1:**
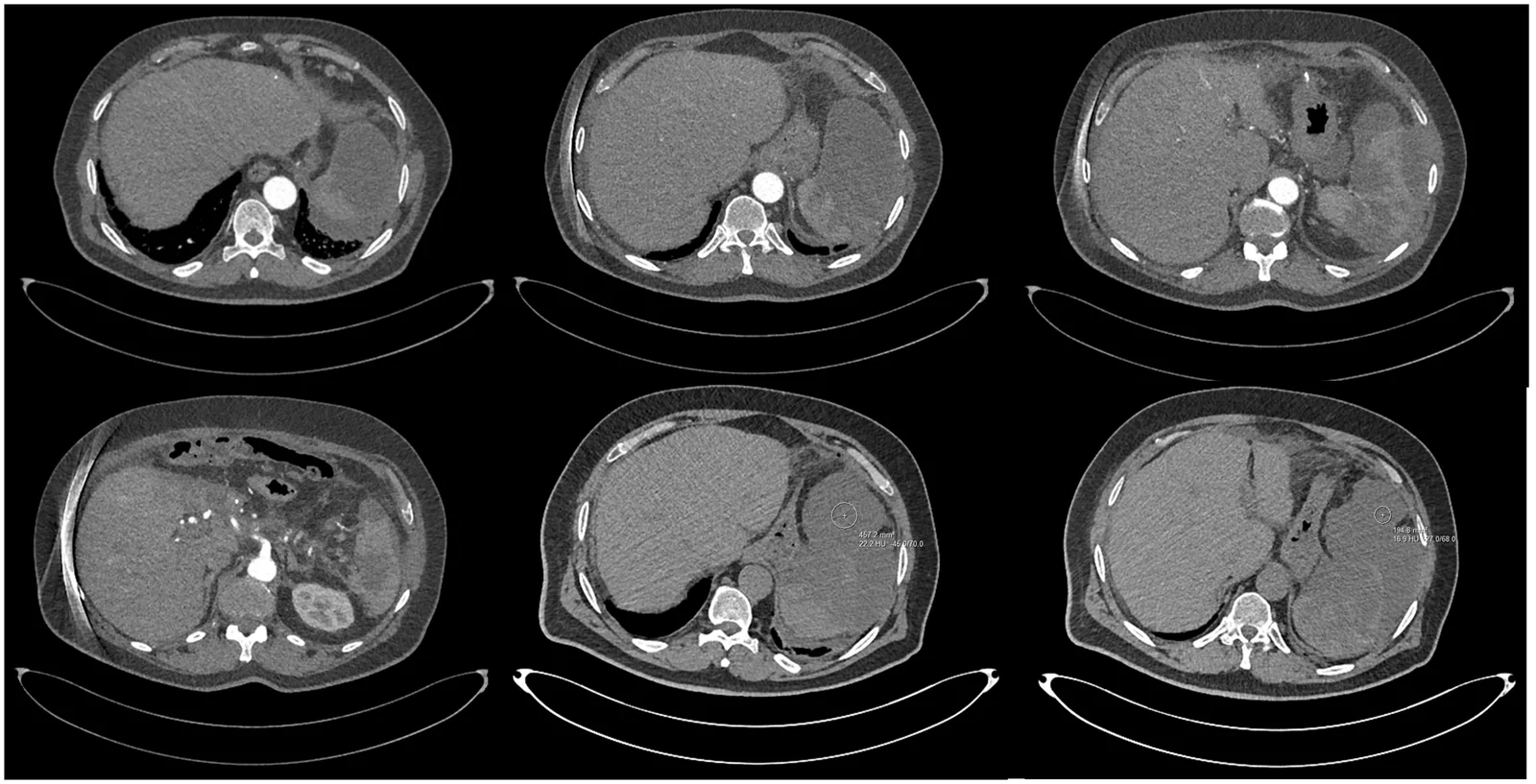
Abdominal CT-scan with and without contrast displaying splenic infractions with upper left quadrant collection.

## Discussion

This case highlights key issues in multidisciplinary PDAC care: the oncologic indication for total duodenopancreatectomy (TDP) in selected isthmo-corporeal tumors; the technical and ischemic risks of spleen-preserving techniques; and the management of postoperative splenic infarction—especially its impact on timing of adjuvant therapy and overall oncologic prognosis.

TDP is an uncommon but defensible oncologic strategy when tumor location and extent make segmental resection unlikely to secure durable local control or an R0 margin [2, 3]. Our patient’s isthmo-corporeal PDAC underwent neoadjuvant therapy and intraoperative assessment indicated spleen-preserving TDP to maximize resectability. Final pathology confirmed residual moderately differentiated ductal carcinoma with high-grade PanIN, CAP TRG 2 response, and multiple adverse features—lymphovascular emboli, perineural invasion, and peripancreatic fat infiltration—together with a high nodal burden (6/26 positive, largest 7 mm, one capsular breach) and ypT3 N2 Mx staging. These findings indicate persistent biologic aggressiveness despite neoadjuvant therapy and support the need for prompt consideration of adjuvant systemic therapy tailored to the patient’s performance status and comorbidities [1].

Spleen preservation aims to reduce hematologic and infectious morbidity associated with splenectomy [4]. Indeed, multiple series report lower rates of postoperative infectious complications and reduced long-term risk of overwhelming post-splenectomy infection and thrombotic events when the spleen is preserved, as well as improved hematologic and immunologic profiles; however, the absolute benefit varies between cohorts and depends on patient selection and surgical technique. However, vessel-preserving and Warshaw-type techniques are technically demanding and may compromise splenic perfusion if short gastric or collateral vessels are injured or insufficient [4, 5].

The postoperative splenic infarct—presenting with left upper-quadrant pain radiating to the scapula and managed non-operatively with analgesia and oral antibiotics—illustrates that spleen preservation carries a measurable ischemic risk. Practical implications include strict intraoperative attention to vascular pedicles, routine consideration of intraoperative perfusion assessment (visual inspection, Doppler, or ICG angiography when available), clear thresholds for postoperative imaging (contrast CT/CTA) when perfusion is suspected, and preplanned vaccination/prophylaxis pathways if splenic function is lost [5, 6].

In this patient the postoperative left-upper-quadrant pain and CT-confirmed focal splenic infarction were managed non-operatively with analgesia and a short course of oral antibiotics; inflammatory markers fell and no abscess, hemorrhage, or hemodynamic compromise occurred, supporting continued conservative management and preservation of splenic function. [6] If splenic function is ultimately lost or splenectomy required, follow asplenia vaccination and prophylaxis guidelines (pneumococcal, meningococcal, *Haemophilus influenzae*) and document plans for antibiotic prophylaxis/education.

## Conclusion

Spleen-preserving TDP can achieve margin-negative resection for selected isthmo-corporeal PDAC with adjacent-organ invasion, but it carries distinct ischemic and metabolic risks. Unexpected granulomatous nodal inflammation mandates a prompt and thorough etiologic workup because the result can change adjuvant management. Clear reporting of staging, technical details, postoperative management, and diagnostic follow-up is required to inform clinical decision-making and to place single cases in the appropriate context of oncologic safety and patient-centered outcomes.

## Highlights

- Laparoscopic spleen-preserving total duodenopancreatectomy (TDP) achieved an R0 resection for an isthmo-corporeal pancreatic ductal adenocarcinoma following neoadjuvant therapy.
- Postoperative splenic infarction is a recognized complication of spleen-preserving approaches that can be successfully managed conservatively without splenectomy.
